# Communicating and Monitoring Surveillance and Response Activities for Malaria Elimination: China's “1-3-7” Strategy

**DOI:** 10.1371/journal.pmed.1001642

**Published:** 2014-05-13

**Authors:** Jun Cao, Hugh J. W. Sturrock, Chris Cotter, Shuisen Zhou, Huayun Zhou, Yaobao Liu, Linhua Tang, Roly D. Gosling, Richard G. A. Feachem, Qi Gao

**Affiliations:** 1 Jiangsu Institute of Parasitic Diseases, Wuxi, China; 2 Key Laboratory of Parasitic Disease Control and Prevention, Ministry of Health, Wuxi, China; 3 Jiangsu Provincial Key Laboratory of Parasite Molecular Biology, Wuxi, China; 4 Global Health Group, University of California, San Francisco, San Francisco, California, United States of America; 5 National Institute of Parasitic Diseases, Chinese Center for Disease Control and Prevention, Shanghai, China; PLOS Medicine, United Kingdom

## Abstract

Qi Gao and colleagues describe China's 1-3-7 strategy for eliminating malaria: reporting of malaria cases within one day, their confirmation and investigation within three days, and the appropriate public health response to prevent further transmission within seven days.

**Summary Points**

- Malaria elimination requires surveillance systems that can reliably rapidly detect and respond to individual cases.
- China's “1-3-7” approach defines targets used to guide and monitor case reporting, investigation, and response, respectively: reporting of malaria cases within one day, their confirmation and investigation within three days, and the appropriate public health response to prevent further transmission within seven days.
- Central to the value and effectiveness of the approach is the ability to communicate information between administrative levels to encourage rapid and complete reporting as well as improved adherence to surveillance and response procedures by health personnel.
- The 1-3-7 approach is a valuable and simple set of targets that could be adopted by other countries and by similar disease elimination programs.

## Introduction

Taking a malaria control program from the control phases through to elimination is challenging. The reorientation of the malaria control program involves a shift of focus, from reaching high levels of coverage of interventions to prevent morbidity and mortality, to an emphasis on completeness and timeliness of activities in order to seek out infections and interrupt transmission. The need to communicate this change in strategic thinking to large, cumbersome health systems has proven a challenge. Has China found a solution?

**Figure 1:**
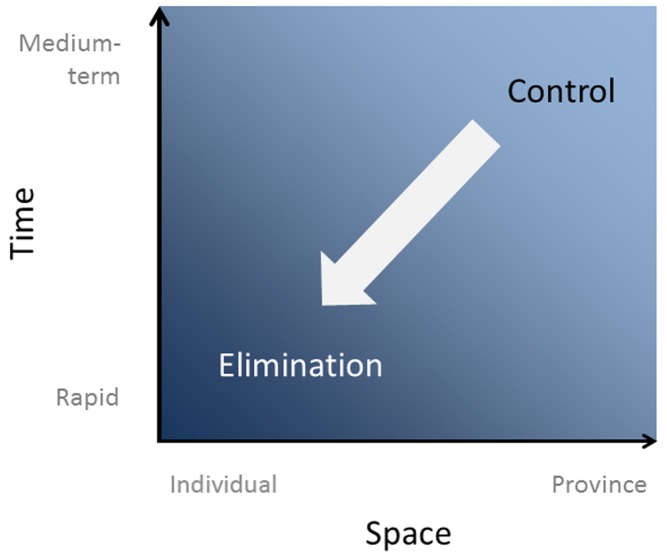
Shifts in the spatial and temporal scales of reporting and response as countries progress towards malaria elimination.

Surveillance to rapidly identify potential and ongoing areas of disease transmission that initiates a rapid and high-quality targeted response is essential for any disease elimination program. For malaria elimination programs, surveillance and the ensuing response includes refinement in both the spatial aspects of reporting and the timeliness of activities (see Figure 1). Malaria programs in countries that have regions with low endemicity or that are already on a path to malaria elimination operationalize the need to rapidly identify infections and prevent them from spreading through a strategy of surveillance and response. The most widely adopted surveillance and response approach is a strategy called reactive case detection (RACD), whereby household members, neighbors, and other contacts of passively detected cases are screened for infection and treated with antimalarials [1]–[4]. The RACD response is often combined with other essential components of an elimination campaign, namely, vector control and community education and participation. The RACD process is similar to contact tracing in tuberculosis and relies on the fact that at low transmission levels, malaria is highly clustered geographically into micro-foci, or hotspots, or is clustered demographically into high-risk groups, or “hot populations” [2],[5]. Although the effectiveness of RACD in reducing malaria transmission has not been demonstrated, RACD is a widely adopted and implemented malaria elimination strategy. Thirteen out of 14 countries in the Asia Pacific Malaria Elimination Network [6], several countries in Africa (implemented in Swaziland [3] and South Africa [7], and in pilot programs in Zambia [8], Senegal [9], and Namibia), and countries in the elimination phase globally employ some form of RACD activity. RACD programs that have carried out monitoring and evaluation of their systems find that there are challenges with operationalizing RACD, particularly with regards to the timely completion of activities and reaching satisfactory coverage of the target population [3]. At the last stages of malaria elimination, programs must be highly vigilant and seek to find every infection. When identified, action to prevent onward transmission of malaria must be complete and prompt. In health systems that rarely see malaria, as is common in settings close to malaria elimination, communicating to health workers the need to urgently and accurately report cases and carry out RACD, and the need to monitor their actions, is problematic. Here we describe the 1-3-7 system employed by the national malaria elimination program in China to communicate and monitor its key malaria elimination strategy.

China launched its malaria elimination program in July 2010 with a plan to achieve elimination by 2020 [10]. Malaria cases (local and imported) have been reduced from more than 26,000 in 2008 to 2,716 in 2012, of which only 243 were due to local transmission. *Plasmodium falciparum* has been almost eliminated (only 16 cases of falciparum malaria in 2012, along the China–Myanmar border) [11]. This success has been driven by a focused program delivering and monitoring targeted interventions to those at risk, including a RACD program that is described by 1-3-7 (see Box 1).

**Box 1. The 1-3-7 Strategy Designed to Guide and Monitor Malaria Surveillance and Response in China**

**1**: Case reporting within one day. Any confirmed and suspected malaria cases by law must be reported to the web-based health information system within 24 hours of diagnosis by the local health-care provider.

**3**: Case investigation within three days. All malaria cases should be confirmed and visited by the county-level China CDC, where the case is reported within three days, to determine where the case originated (local or imported).

**7**: Focus investigation and action within seven days. The focus investigation should be conducted as soon as possible. If local transmission is possible or confirmed, targeted action to seek out other infections and reduce the chance of onward transmission is completed within seven days by the county-level China CDC of the county where the patient resides and/or works.

## Communicating the Strategy

1-3-7 is a simplified set of targets that delineates responsibilities, actions, and their time frame. These specific targets were set following discussions with the Ministry of Health, malaria experts, and local health workers, and were deemed feasible based on national and local capacity.

## Case Reporting within One Day

**Figure 2:**
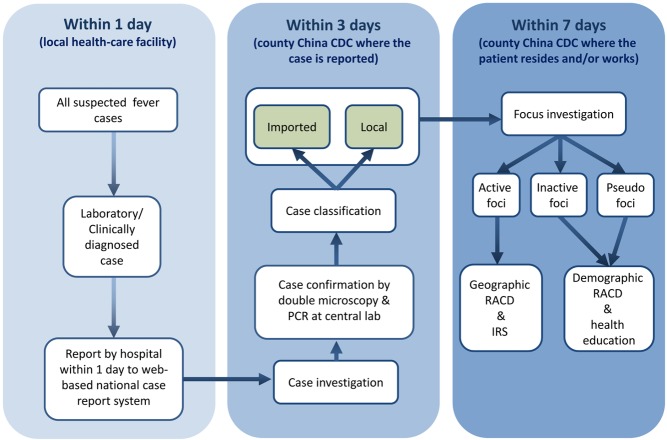
Schematic of the chain of events conducted within the 1-3-7 time windows. IRS, indoor residual spraying.

**Figure 3:**
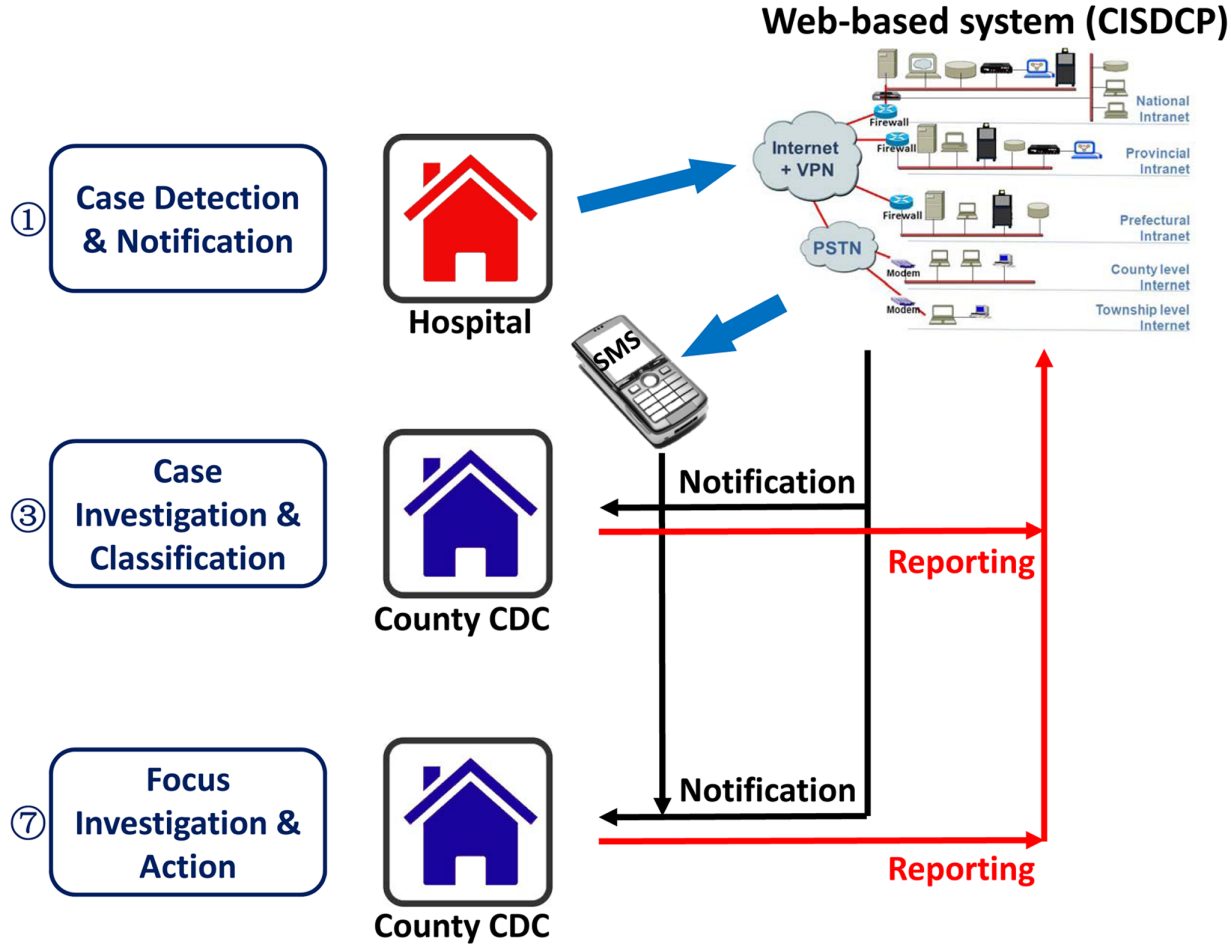
Diagram of the data reporting and feedback system for 1-3-7. CDC, China CDC; PSTN, public switched telephone network; SMS, short message service; VPN, virtual private network.

All individuals with fever who present at local health-care providers are tested for malaria parasites using microscopy or a rapid diagnosis test (RDT), with those positive treated immediately with antimalarials according to China's drug policy (Figure 2). Because malaria is now rare in China, the quality of malaria diagnosis cannot be assured at lower levels of the health system. Thus, local health-care providers are encouraged to report both confirmed and suspected malaria cases within 24 hours using the web-based China Information System for Disease Control and Prevention (CISDCP) [12]. A cellphone-based short message service alert system immediately informs local Chinese Center for Disease Control and Prevention (China CDC) staff of a case, ensuring timely follow-up (Figure 3).

## Case Investigation within Three Days

 Case investigation comprises two components that should both be completed within three days: (1) case confirmation and (2) case classification. Cases are confirmed via double reading of slides by expert microscopists, and where possible quality assured using PCR at the provincial laboratory, albeit with some delay. Cases are classified as being locally acquired or imported by a careful epidemiological investigation according to China's technical scheme of malaria elimination [13]. A case is classified as imported only if the individual has travelled to a malaria-endemic country within the previous month, or has travelled to another district in China with clear evidence of malaria transmission (local cases reported). Otherwise, the case is classified as local. If the origin of the infection is difficult to determine, a provincial or national expert team will review the case and ascertain origin.

## Focus Investigation and Action within Seven Days

Irrespective of whether the case is classified as local or imported, the local area around a case (the focus) is investigated to evaluate the risk of local transmission. Different actions are triggered according to the results of the investigation and should be completed within seven days. If the local area is not thought to support transmission because of the absence of vectors (i.e., during the non-transmission season or without a capable vector—*Anopheles sinensis* is unlikely to transmit *Plasmodium falciparum*), the focus is classified as an “inactive focus.” If the case is classified as imported and resides in a malaria-free area, the focus is classified as a “pseudo focus.” For both inactive and pseudo foci, RACD screening is carried out in demographic contacts of the case (“hot populations”) such as coworkers who travelled to the same area, and health education materials are distributed. Where viable vectors are identified, the focus is classified as an “active focus.” For active foci, vector control (indoor residual spraying) and more intensive RACD (up to 200 neighbors screened) is initiated. RACD is conducted using RDTs for immediate results, with filter paper blood spots collected from all people screened and taken to the provincial laboratory for later molecular (PCR) testing in order to detect low-density infections that would be missed by RDTs.

## 1-3-7 Reporting

For communication purposes, summary statistics of the three indicators (i.e., proportion of cases reported in one day, investigated in three days, and responded to in seven days) are produced by the CISDCP and fed back to a number of administrative levels (Figure 3). Daily data are collated and verified at the county level by the county-level China CDC staff, who follow up the individual cases and the health facilities that reported the case. At the provincial level, a weekly case analysis report is produced by the Provincial Malaria Elimination Program Manager; this report includes a brief investigation report for each case. Monthly 1-3-7 implementation analysis reports are generated by the Ministry of Health and circulated to all Provincial Health Departments and at a national level to the National Institute of Parasitic Diseases for monitoring and evaluation purposes. Once the case investigation or response activity for a case is complete, there is a reporting channel that feeds into the larger information system to provide critical feedback on the results of the response and action for each index case. Many disease control programs yearn for such a feedback loop, whereby all levels of the system are reminded of their responsibilities and can monitor their own performance.

## China's Progress

Following the launch of the National Malaria Elimination Program in July 2010 [9], the China CDC issued *China's Technical Scheme of Malaria Elimination* in September 2011 [13], which identified the key activities for malaria surveillance and response during the elimination stage and the time frame of expected activities. The 1-3-7 strategy was rolled out nationally in early 2012. In July 2012, the Ministry of Health initiated monthly reports for wider dissemination. In October 2012, the monthly reporting was integrated into the web-based health information system (CISDCP).

**Figure 4:**
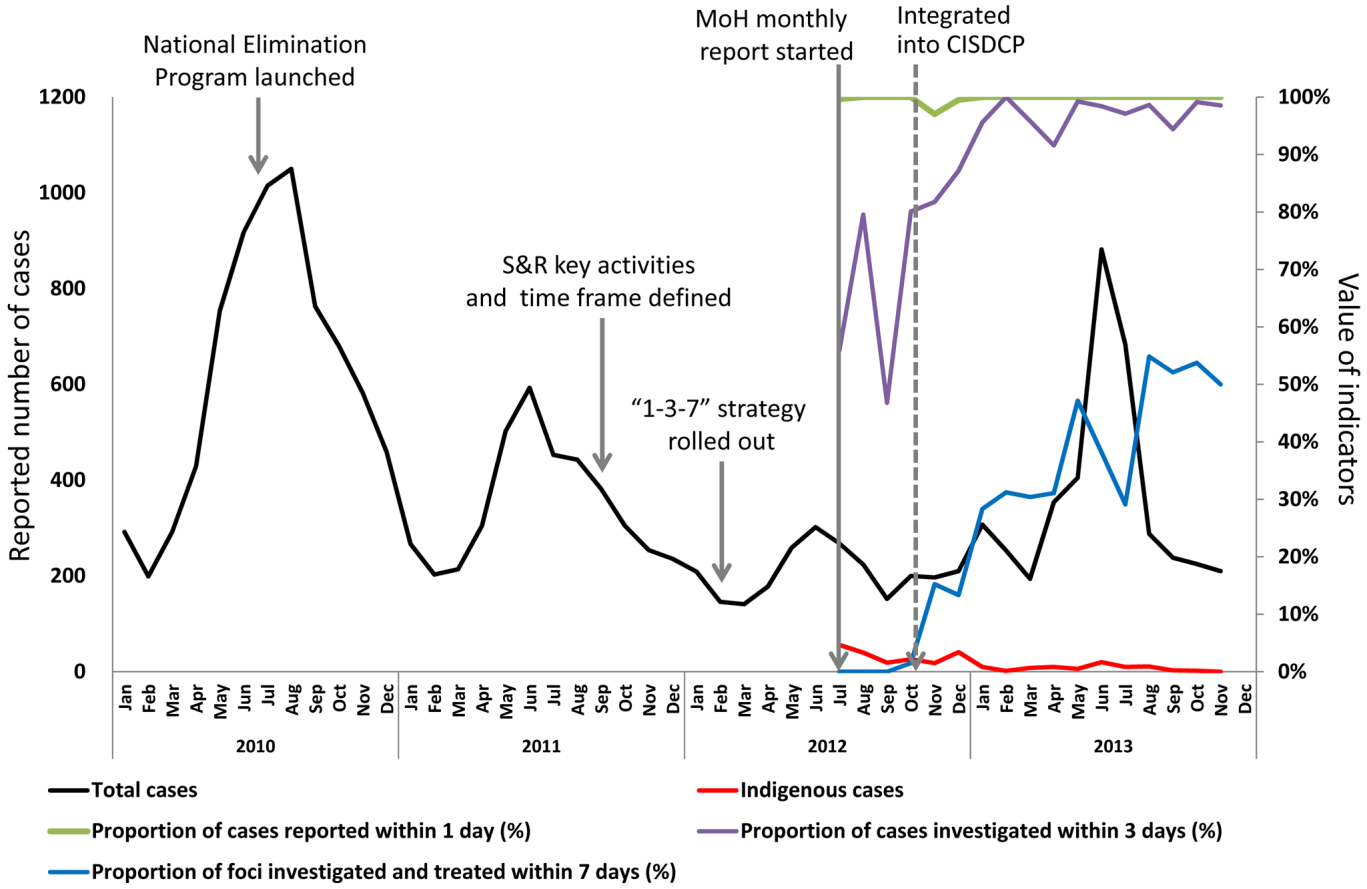
Implementation progress of the 1-3-7 strategy in China. MoH, Ministry of Health; S&R, surveillance and response.

After the initiation of monthly reports by the Ministry of Health in July 2012, the timeliness of implementation of malaria surveillance and response significantly improved, most notably for case investigation. Case reporting is almost always done within one day because this is the timeline required by law. Throughout the remainder of 2012 and 2013, the proportion of cases investigated within three days increased substantially from around 55% to the nearly 100% seen today (Figure 4). While there have been improvements in the timeliness of focus investigation and response, the proportion of cases for which this occurs within seven days still requires improvement.

## Challenges

Central to the widespread adoption of the 1-3-7 approach is the real-time reporting of case data. Achieving such rapid reporting required overcoming two major challenges: developing an information technology system to handle the flow and storage of case data, and ensuring that the system is effectively used by health personnel. The information technology challenge was overcome in China by integrating malaria reporting into the web-based health information system (CISDCP) established after the severe acute respiratory syndrome outbreak. Once the system was established, encouraging health staff to move from paper-based reporting to using the CISDCP was not always a simple process. To address this issue, the Ministry of Health introduced a small financial incentive. For example, in Jiangsu Province workers are paid 30 CNY (US$ 4.80) for reporting, and 70 CNY (US$ 11.30) for completing case investigation. This incentive to compensate for the additional work required has led to substantial improvements in the use of the system and remains in place to encourage adherence.

Moving forward, key challenges affecting the 1-3-7 approach remain. First, PCR confirmation of all cases within the three-day target and screening of household members, neighbors, and networks of cases within the seven-day target are continuing challenges. Such PCR confirmation is vital given the importance and prevalence of sub-microscopic infections [14]. Confirming the initial diagnosis ensures that any initial false negatives can be followed up and that false positives do not initiate an unnecessary response. PCR confirmation of the household and neighbor screening samples ensures that low-density infections are detected. The process of testing samples can be challenging: transporting and processing samples often takes longer than three days, and follow-up several days later can result in difficulties retracing cases. Efforts have been made to improve transport logistics to support immediate testing of blood spots at central laboratories. In addition, sensitive point-of-care molecular tests, such as loop-mediated isothermal amplification, are being developed [15]. Second, better methods are required to classify the source of infection. Currently, case classification is made based on travel history alone, which may lead to misclassification, particularly of *P. vivax* cases, which may be due to relapsed or recrudescent infections. Genotyping that can distinguish the geographical origin of infection may improve the designation of these classifications. However, standardized genotyping methods are not yet defined and are currently unlikely to be completed within the three-day window. Third, completion of focus investigation and response within the seven-day window is challenging because of the time required for comprehensive assessment of transmission risk and for planning and implementation of response activities. Hitting the seven-day target is especially difficult during the transmission season, June and July, when locally transmitted case numbers are highest and determination of foci is the most difficult. In order to speed up the process of transmission risk assessment and choice of response package, standard operating procedures are being developed. Further reassessment of data generated through the 1-3-7 system will support whether the seven-day target should be changed. Finally, the human and financial resources needed to reach the 1-3-7 targets may be considerable, and evaluating the cost-effectiveness of the 1-3-7 strategy and other time frame targets may be necessary.

## Conclusions

Communicating and monitoring disease-specific algorithms is essential in elimination campaigns and is particularly challenging when the disease is increasingly rare. The 1-3-7 strategy to encourage timely reporting of malaria cases, their investigation, and appropriate public health responses has a simplicity that is easy to understand, clearly defines roles and time frames, and is measurable. It should be made clear, however, that the 1-3-7 strategy is one to guide and monitor elimination activities rather than a collective term for the activities themselves. The 1-3-7 algorithm will, therefore, be suitable only in elimination settings where investigating and responding to every case is currently implemented or planned for implementation, as is the case in many low-malaria-endemic countries. In higher transmission and some pre-elimination settings, resources are often too limited to investigate and respond to large numbers of cases. A number of countries in the Asia Pacific region, members of the Asia Pacific Malaria Elimination Network, are considering adopting the 1-3-7 algorithm for their own malaria elimination programs. In many instances, the adoption of 1-3-7 will require adjustments to the time frame. For example, in the Solomon Islands, reporting of cases within 24 hours is often impossible because of lack of mobile phone or internet coverage. Reporting of cases via VHF radios stationed at health posts can only be done within 48 hours, meaning a “2-4-7” system may be more appropriate. In addition, a 1-3-7-like approach provides a simple set of targets for programs to employ when assessing new elimination strategies, such as mass drug administration in response to an outbreak. The key component of the algorithm is that both the completeness and timeliness of activities are captured.

Could similar strategies be used to measure other disease elimination programs and be rolled out globally? For malaria elimination there is a dearth of useful indicators; 1-3-7 is certainly operationally useful and, if the correct information system is in place, is easy to measure. If widely adopted, such an indicator system would provide a robust and epidemiologically relevant standard against which to make comparisons and chart the progress of elimination across regions.

## Abbreviations

China CDC: Chinese Center for Disease Control and Prevention
CISDCP: China Information System for Disease Control and Prevention
RACD: reactive case detection
RDT: rapid diagnostic test
